# Energy-Efficient and Economy-Sustainable Technology for Online Seed Viability Detection Using Hyper Spectrum

**DOI:** 10.3390/s26041333

**Published:** 2026-02-19

**Authors:** Xiantao He, Yichen Li, Jinting Zhu, Ceang Wen, Dawei Sun, Li Yang, Dongxing Zhang, Tao Cui, Kailiang Zhang, Ying Deng

**Affiliations:** 1 College of Engineering, China Agricultural University, Beijing 100083, China; hxt@cau.edu.cn (X.H.); liyichen@cau.edu.cn (Y.L.); zhujinting99@163.com (J.Z.); wenca@cau.edu.cn (C.W.); zhangdx@cau.edu.cn (D.Z.); cuitao850919@163.com (T.C.); zhang_kailiang@cau.edu.cn (K.Z.); dengying.xi@foxmail.com (Y.D.); 2 Institute of Agricultural Equipment, Zhejiang Academy of Agricultural Sciences, Hangzhou 310000, China; dzs0015@tigermail.auburn.edu

**Keywords:** mung bean, energy-efficient, economy-sustainable, hyper spectrum, seed viability detection

## Abstract

The seed viability detection before sowing is indispensable in the agricultural production of mung beans. The conventional detection methods for seed viability are destructive, carry a risk of contamination, and fail to identify individual non-viable seeds. In this study, an efficient and sustainable method for online viability detection of mung bean seeds was developed, which utilized hyperspectral techniques and had characteristics of rapid speed, non-destructive analysis, and the ability to detect the viability status without pollution to the environment. A sample holder for mung bean seeds was designed to stably collect spectral data. The effects of different optimal spectral bands and modeling algorithms on the detection accuracy of seed viability were analyzed. Compared to the support vector machine (SVM) and the extreme learning machine (ELM) algorithms, the partial least squares (PLS) algorithm based on the visible and near-infrared spectra (380~980 nm) had better performance. The accuracy for the identification of non-viable seeds was 98.8%, and the error of viability prediction was 20.71%. The cost of a one-time viability test is $0.25 with energy consumption of 0.05 kWh^−1^, which is much lower than the germination test with a cost of $80.2 and energy consumption of 50.4 kWh^−1^. Furthermore, individual non-viable seeds can be identified and removed, and the revenue increases by $286.9 per hectare after sorting the non-viable seeds from the seeds with an 85% germination rate. This will promote the cleaner production of mung beans without additional chemical solutions added in the process.

## 1. Introduction

Mung bean is rich in nutrients, with characteristics of high protein, medium starch, low fat, and a variety of vitamins and minerals. It is widely grown and has been regarded as an important nutritional health care product and food medicine [1,2,3]. The seed viability is one of the most important factors affecting the emergence rate and seedling growth, thereby seriously affecting crop yield [4,5,6,7]. However, a variety of external environmental factors can cause a decrease in seed viability or even cause it to lose its viability, such as inappropriate harvest time [8,9], high drying temperature [10,11,12], unsuitable ambient temperature and humidity [13,14,15], and diseases and insect pests during storage [16,17]. Therefore, the seed viability test is indispensable in the agricultural production of mung beans.

Currently, most viability testing methods for mung bean seeds are destructive or carry a contamination risk and fail to identify individual non-viable seeds. The germination test [18,19] is the most commonly used method to measure the viability of mung bean seeds, but it is labor- and time-consuming, energy-wasting, and destructive. The tetrazolium test [20,21], the bromothymol blue method, the red ink test, and the conductivity method [22] are commonly used methods for rapid prediction of seed viability level during mung bean production. These methods determine the overall seed viability by measuring the degree of damage to the physiological and metabolic functions of seeds. However, these methods produce corresponding acidic and alkaline chemicals and carry the risk of pollution to water and soil. Therefore, there is an urgent need to develop a rapid, non-destructive, green, and low-consumption method for the viability detection of individual mung bean seeds, thus achieving efficient and clean production of mung beans.

Hyperspectral techniques have been widely used in agricultural production because of their advantages of fast detection and non-destructiveness [23,24,25,26,27], especially in the rapid acquisition of seed physicochemical information [28,29,30]. For instance, hyperspectral techniques can be used not only for quantitative analysis to determine the protein [31,32], oil [32,33], carbohydrate [34], and moisture content [35] of seeds, but also for the qualitative analysis to identify seed variety [36], origin [37], crop year [38] and quality [39]. Furthermore, hyperspectral techniques have also been gradually applied in the nondestructive detection of seed viability. Based on the techniques, researchers have explored the feasibility of detecting the seed viability of several staple crops such as maize [40,41,42], rice [43,44] wheat [45,46], etc., and vegetables and fruits, such as tomato [47], watermelon [48], melon [49], retinspora [50], pepper [51], etc., which shows that the hyperspectral techniques have great potential in the detection of seed viability. Nevertheless, there are few reports on the application of hyperspectral techniques to the viability detection of mung bean seeds, and there is no equipment designed for seed viability detection and non-viable seed screening, which restricts the efficient and sustainable development of the mung bean industry.

A spectrometer and a hyperspectral imager are two main hyperspectral devices. The spectrometer only collects spectral information [52,53], which contains average spectral information for a certain region of the sample and is mainly aimed at organic content detection of liquids, powders, or homogeneous solids [54,55,56]. A hyperspectral imager has an image at each band, thus making it capable of acquiring both spectral information and image information [29,57]. However, the data collection time and cost of a hyperspectral imager are much higher than those of a spectrometer [58]. Therefore, this study chose the spectrometer to collect spectral data of mung bean seeds.

Despite these advances, significant gaps remain regarding the application of spectral methods to minor crops like mung beans, specifically the lack of individual seed identification capability in existing non-destructive methods and the absence of systematic evaluation of economic sustainability and real-time applicability. The objectives of this study are as follows: (1) to develop a rapid, non-destructive, and environmentally friendly method for detecting mung bean seed viability using Vis/NIR spectroscopy; (2) to design a real-time detection and sorting platform for individual non-viable seed removal; and (3) to evaluate the economic sustainability and practical applicability of the developed system compared with conventional methods. This study addresses the urgent need for cleaner production technologies in mung bean agriculture while providing a solution for seed quality control.

## 2. Materials and Methods

### 2.1. Samples and Sample Preparation

This study collected 12 varieties of mung bean seeds from seven regions (covering major mung bean-producing areas of China), which were harvested in 2021 and generously provided by the China Agriculture Research System of the Ministry of Finance (MOF). The detailed information on the seeds is shown in Table 1. Before the experiment, all collected mung bean seeds were sealed in glass jars and then stored in a constant temperature refrigerator at 5 °C to eliminate the effects of ambient temperature and air humidity on seed viability. To simulate the natural viability loss process and ensure experimental consistency and repeatability, we adopted the microwave heating method, which has been widely used in seed viability studies [41,42]. Microwave heating induces protein denaturation, starch gelatinization, and damages the membrane system, which is similar to the physiological changes occurring in naturally aged seeds. The microwave power was 700 W and the time was 40s, which was determined by experiments to ensure that the mung bean seeds completely lost their vitality. In total, 400 seeds of each variety were taken, of which 200 were inactivated by the microwave heating method, and the other 200 seeds were used as the control group. As a result, a total of 4800 samples were obtained, including 2400 normal seeds and 2400 non-viable seeds. Conventional modeling methods generally divide a data set into a trained class and a predicted class, which could achieve remarkable performances, whereas an independent predicted class could better evaluate the modeling approaches. Therefore, 8 of the 12 seed varieties (numbers 1 to 8 in Table 1) were used as the trained class, and the remaining 4 independent varieties (numbers 9 to 12 in Table 1) were used as the predicted class.

### 2.2. The Design of the Sample Holder and Spectral Data Acquisition

**Figure 1:**
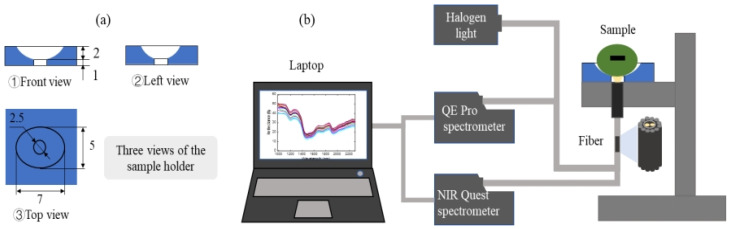
(**a**) The shape and size of the designed sample holder and (**b**) the spectrum acquisition platform for mung bean seeds.

The length and width of single mung bean seeds were measured with a slide caliper (Deli group Co., Ltd., Ningbo, China). The length (mm) of the mung bean seed is the longest length of the kernel, and the widest width of the kernel is treated as the width (mm) of the mung bean seed. All varieties were measured in three replications. The length and width of all mung bean seeds (12 varieties with a total of 120 seeds) ranged from 3.79 to 6.51 mm and 3.07 to 4.82 mm, respectively, as shown in Table 1. Considering the size range of the mung bean, a sample holder with a semi-ellipsoidal aperture was designed to meet the different size requirements. The major axis and minor axis of the top elliptic section were sized 7 nm and 5 nm, respectively, with 2 mm to the bottom of the ellipsoid (Figure 1a). Thus, the mung bean samples could be accommodated in the holder and, as a result, stable spectral data could be collected. The central portion of the holder was made of round glass (diameter 2.5 mm, thickness 1 mm) for good transmission of light in the visible (Vis) and near-infrared (NIR) regions. The reflection methods for mung bean seeds were selected, and an explanatory diagram of the spectral acquisition platform with the holder is shown in Figure 1b.

The spectrum acquisition platform included two spectrometers, i.e., QE Pro and NIR Quest (Ocean Optics, Inc., Orlando, FL, USA), a HL-2000 halogen lamp (Ocean Optics, Inc., Orlando, FL, USA), a trigeminal optical fiber, a computer with Ocean View software (Ocean Optics, Inc., Orlando, FL, USA), and a sample holder. The selection of spectrometers over a hyperspectral imager was driven by practical considerations, as they offered a more suitable solution for the rapid and cost-effective viability assessment of individual seeds. The spectral range of the spectrometer QE Pro is 185~1100 nm with a resolution of 1.7 nm, which includes the Vis region (380~780 nm) and part of the NIR region (780~1100 nm). The spectrometer NIR Quest covers most of the NIR spectrum (900~2500 nm with a resolution of 9.0 nm). Several parts of spectral data (185~380 nm, 980~1100 nm for QE Pro, 900~980 nm, and 2298~2500 nm for NIR Quest) were omitted because of a low signal-to-noise ratio, and the spectral ranges retained for QE Pro and NIR Quest were 380~980 nm (Vis/NIR) and 980~2298 nm (NIR), respectively. To meet the requirements of real-time spectrum acquisition, the integration time of the two spectrometers was set to 0.068 s. The power of the halogen lamp was 5 W, and it could continuously provide stable light for the operation.

The fiber probe was fixed beneath the sample holder with a hole in the center to reduce the influence of ambient light during the collection of spectral data. The mounting distance of the fiber probe (the distance between the fiber probe and the sample holder) was 0.5 mm, so that the field of view was close to 2.5 mm in diameter. The three splices of the optical fiber were connected to the two spectrometers and the halogen lamp, respectively. The total number of fiber cores was 14, including 12 outer cores and 2 inner ones. All outer cores were connected to the halogen lamp for a uniform light source, while the inner cores were connected to the QE Pro and NIR Quest, respectively, for the simultaneous spectral data collection.

### 2.3. Microscopic Examination and Germination Test of Seed

The microstructures of normal and non-viable seeds were examined by a Leica DVM6 electron microscope equipped with LAS X software (Leica Microsystems, Inc., Wetzlar, Germany). An objective lens of FOV 3.60 was selected, and then the mode of reflection microscopy was set to obtain high-magnification microscopic images of the seed section. The microscopic images of the full-view seed embryo, embryonic cell, and endosperm cell were collected with 70, 400, and 400 magnifications, respectively.

The viability of all seeds was determined by the seed germination test [22]. The two filter papers served as a germination bed to provide moisture for the seeds and support the growth of the seedlings, and they were placed in a transparent and non-toxic germination container. Each germination bed was evenly populated with 100 mung bean seeds, and the seed spacing was about twice the diameter of the seeds to provide enough space for the seedlings. The germination test was conducted in an artificial climate chamber (Ningbo Ledian Instrument Manufacturing Co., Ltd., Ningbo, China) at 25 °C and 12,000 xl for 7 days. Seeds with a 2 mm seedling were considered viable; otherwise, they were considered non-viable. The viability index (*VI*) was calculated as the product of germination rate and average seedling length:

*VI* = *G* · *S* (1)

where *G* is the germination rate (%) divided by 100, and *S* is the average seedling length (cm). This formula integrates both the germination capacity and seedling vigor, providing a comprehensive measure of seed viability.

### 2.4. Classification Models

The collected spectral data were preprocessed by the Savitzky–Golay smoothing (SG) algorithm to eliminate high-frequency noise in the spectrum with a sliding smoothness width of 5. Three machine learning methods, i.e., the partial least squares (PLS), the support vector machine (SVM), and the extreme learning machine (ELM) algorithms, were selected for real-time seed vigor prediction. PLS is a frequently used multivariate statistical analysis method and is particularly suited when there is multicollinearity among spectral data (matrix *X*). PLS is a latent variable method to form a covariance model in the two spaces (matrices *X* and *Y*) and determines the multi-dimensional direction of the *X* space that expresses the maximum multi-dimensional variance direction of the *Y* space, which is calculated by the following formula.

(2) $$X=TU^{T}+E$$

(3) $$Y=PV^{T}+F$$

where *X* is an *m* × *n* matrix of prediction, and *Y* is an *m* × 1 matrix of response. *T* and *P* are the score matrices, while *U* and *V* are the loading matrices. Matrices *E* and *F* are the idiosyncratic errors. In the model construction stage, the number of principal components was optimized by the method of 10-fold cross-validation, and 12 principal components were finally determined.

SVM is a high-performance and well-known machine learning model for solving nonlinear and high-dimensional data classification problems. The SVM algorithm is characterized by maximal margins, high-dimensional mapping, and kernel methods. Among them, high-dimensional mapping is the key to solving nonlinear classification problems with linear methods and can map low-dimensional inseparable or nonlinear data to a high-dimensional space to achieve linear separability. The kernel function selected in this study is the Gaussian kernel function (Gaussian kernel), which is very suitable for processing nonlinear or linear data with high flexibility.

The ELM algorithm is a fast-learning method. The model parameters, such as weights and thresholds of the input as well as hidden and output layers, are obtained by the Moore–Penrose method based on the least squares criterion. The complex classification problem can be transformed into a linear system solution problem by ELM, which has the characteristics of quick learning ability and wide suitability, and can meet the real-time requirements of seed vitality detection and screening. The number of hidden neurons assigned to the ELM was set to 2000, and the activation function was a sigmoidal function.

All calculations for data processing and model building were conducted by MATLAB R2018b.

### 2.5. Evaluation Indices

The model performance for classifying non-viable seeds was assessed by accuracy, precision, and recall. Accuracy is an overall index that describes the proportion of correctly recognized seeds in all seeds and is used to comprehensively evaluate model performance. Precision and recall are two supplementary indices. Precision is the proportion of correctly recognized seeds in the predicted class, and recall is the proportion of correctly recognized seeds in the true class. The calculation formulas are as follows.

(4) $$Accuracy=\frac{TP+TN}{TP+TN+FP+FN}\times 100\%$$

(5) $$Precision=\frac{TP}{TP+FP}\times 100\%$$

(6) $$Recall=\frac{TP}{TP+FN}\times 100\%$$

where *TP* and *FN* are true positives and false negatives, respectively. *FP* and *TN* are false positives and true negatives, respectively. The model performance for predicting seed viability was evaluated by the coefficient of determination (*R*^2^), root mean square error of prediction (*RMSEP*), and prediction deviation of seed viability (*PDSV*), which are calculated in Equations (6)–(8).

(7) $$R^{2}=1-\frac{\sum_{i=1}^{n}{\left(y_{i}-{\hat{y}}_{i}\right)}^{2}}{\sum_{i=1}^{n}{\left(y_{i}-\bar{y}\right)}^{2}}$$

(8) $$RMSEP=\sqrt{\frac{1}{n}\cdot \sum_{i=1}^{n}{\left(y_{i}-{\hat{y}}_{i}\right)}^{2}}\;$$

(9) $$PDSV=\frac{AV-PV}{AV}\times 100\%\;$$

where *y*_i_ is the observed value, and $\bar{y}$ is the average of observed values. ${\hat{y}}_{i}$ is the predicted value of the regression model. *AV* and *PV* are actual viability and predicted viability, respectively.

## 3. Results and Discussion

### 3.1. The Microstructure and Viability Status of the Samples

**Figure 2:**
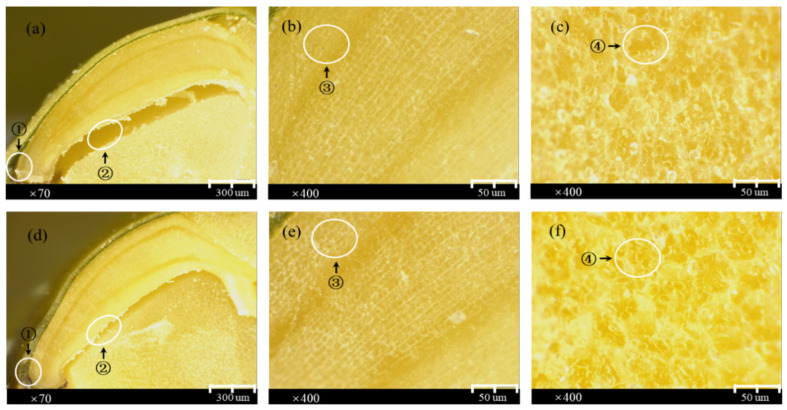
Electron microscope images from different parts of mung bean seeds (Take variety JiLv2 as an example): (**a**) full-view embryo, (**b**) embryonic cell, and (**c**) endosperm cell of non-viable group; (**d**) full-view embryo, (**e**) embryonic cell, and (**f**) endosperm cell of normal group.

Figure 2 shows a microscopic image of different parts of mung bean seeds (full-view embryo, embryonic and endosperm cells) collected by an electron microscope. The gaps between the seed coat and the seed embryo and between the seed embryo and endosperm were significantly larger in the non-viable class than in the normal class (labels 1 and 2 in Figure 2a and Figure 2d, respectively). The cellular structure of the seed embryo in the non-viable class was partly destroyed, and the cell contour was dim (label 3 in Figure 2b), while the cellular structure of the seed embryo in the normal class was intact, and its cell contour was clear (label 3 in Figure 2e). It was indicated that the physiological function of seeds in the non-viable class had been damaged. The cells in the endosperm of the non-viable class were closely packed, which went against the transfer of nutritive material. In contrast, the cells in the endosperm of the normal class were more intact and had gaps between adjacent cells; thus, the nutrition could be transported normally. These differences are the main causes for the identification of non-viable seeds by the spectral method.

The electron microscope images indicated that the cellular structure and function of the seeds in the non-viable class were destroyed, which is in accord with the results acquired from the germination test, with the germination rate of 0%. However, there was no regular difference between the microscopic images of 12 varieties of mung seeds for either the group of normal seeds or the group of inactivated seeds. In addition, viability differences between the 12 normal groups were observed, and the results of the germination test are shown in Table 2. It could be seen that their seed germination rates were all higher than 91%, meeting the index of germination rate for sowing (≥85%). Furthermore, the high seed germination rate is the basis for obtaining high seed viability, and the seeds with a higher germination rate are more likely to obtain high seed vigor (for instance, the highest germination rate of 98.5% and the highest seed viability of 9.55 were both achieved by LF2).

### 3.2. Spectral Characteristics of the Samples

**Figure 3:**
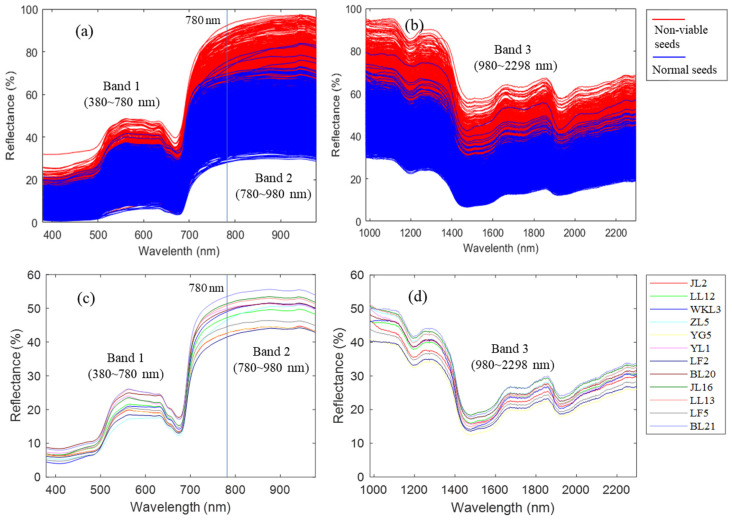
(**a**) Vis/NIR and (**b**) NIR spectra of normal and non-viable seeds; (**c**) Vis/NIR and (**d**) NIR mean spectra of the 12 mung bean varieties.

The spectral data of 12 seed varieties, including normal and non-viable seeds, were analyzed. Despite the similar spectral trends of all seeds, there was a large difference between the normal and non-viable seeds, as shown in Figure 3a,b. The results of the germination test are shown in Table 2, and it was noticed that all seeds treated by the microwave heating method failed to germinate, indicating that these seeds had completely lost their viability. This was because the heat treatment changed the internal structure of the seeds (Figure 2a,b,f), which led to the loss of seed viability and spectral differences between normal and non-viable seeds. It was found that the differences in the two types of seeds in the range of the Vis spectra (band 1: 380~780 nm) could be clearly differentiated by spectrometers, though they were barely distinguished by the human eye. And the spectral differences in the NIR range (band 2: 780~980 nm, band 3: 980~2298 nm) were larger than the differences in the Vis range (band 1: 380~780 nm), especially in the range of band 2. This was because the chemical composition of the mung bean seeds changed (starch gelatinization, protein denaturation, lipid saturation, moisture reduction, etc.) during the inactivation treatment process [40,41]. And less reflectance was presented, meaning more light was absorbed.

Comparing the spectral data collected from the seeds of 12 varieties (Figure 3c,d), the spectral data of each variety had the same trend, but there were differences in spectral reflectance. The higher the seed viability, the lower the spectral reflectance. JL16 had the lowest viability, and LF2 had the highest viability; their corresponding spectral reflectance was the highest and lowest, respectively. Therefore, it is feasible to predict the seed viability of different varieties by using the Vis/NIR or NIR spectrum, and the model with the Vis/NIR spectrum may be more effective in predicting the seed viability due to a larger spectral difference between the varieties. A further evaluation was then conducted to determine the most suitable spectral band for viability detection of mung bean seeds.

### 3.3. The Model for Identifying Non-Viable Mung Bean Seeds

Three algorithms (PLS, SVM, and ELM) were used to build models for identifying non-viable mung bean seeds. The results are shown in Table 3. The highest accuracy of the trained class was achieved by the SVM algorithm, with an accuracy close to 100%. The highest accuracy of the predicted class was obtained by the PLS algorithm, which achieved an accuracy of 98.8% and 97.7% for Vis/NIR and NIR spectra, respectively. The lowest accuracy was observed for the ELM algorithm in this study, with only about 95% accuracy when the NIR spectrum was used. Even though the number of hidden neurons was increased to 2000, the performance was only slightly improved. The PLS algorithm was selected for the identification of non-viable seeds because of its high accuracy and simple structure.

**Figure 4:**
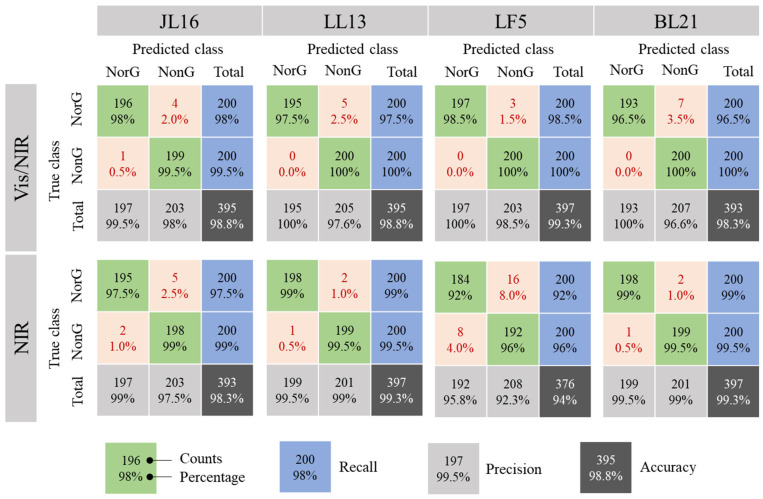
The accuracy for identifying non-viable mung bean seeds by the PLS model with different spectral bands and seed varieties. NorG: normal group, NonG: non-viable group.

The accuracy for identifying non-viable seeds of each variety is shown in Figure 4. The accuracy of the PLS model based on the Vis/NIR spectrum for all four varieties was more than 98%, with an average accuracy of 98.8%, which was better than the accuracy of 97.7% using the NIR spectrum. The spectral resolution of the Vis/NIR spectrometer was 1.7 nm, and the spectral number used for building the model was 803, while the spectral resolution of the NIR spectrometer was 9.0 nm and the spectral number was 412. We speculated that the higher spectral number of the Vis/NIR spectrometer and the large spectral difference in the region of band 2 were the reasons for achieving higher accuracy with Vis/NIR spectroscopy.

Further analysis was conducted, and the identification results of the model built by Vis/NIR and NIR spectra are shown in Figure 4. The PLS model built by the Vis/NIR spectrum could accurately recognize non-viable seeds: only one of 200 non-viable seeds in variety JL16 was mistakenly identified as a normal seed, while all the 200 non-viable seeds of the other three varieties were classified correctly. The PLS model built by the NIR spectrum showed misjudgment in the identification of non-viable and normal seeds in the four varieties. In addition, it was observed that a few seeds in the normal group failed to germinate (Table 2) and were regarded as non-viable (there were seven, seven, six, and seven non-viable seeds in varieties JL16, LL13, LF5, and BL21, respectively). These seeds were mostly identified as non-viable by the Vis/NIR-based model: four of seven, five of seven, three of six, and seven of seven non-viable seeds were identified in varieties JL16, LL13, LF5, and BL21, respectively. Therefore, the Vis/NIR spectrum was preferred for the identification of non-viable seeds, and the resulting PLS model could accurately classify non-viable seeds among normal mung bean seeds.

### 3.4. The Model for Predicting the Viability of Mung Bean Seeds

**Figure 5:**
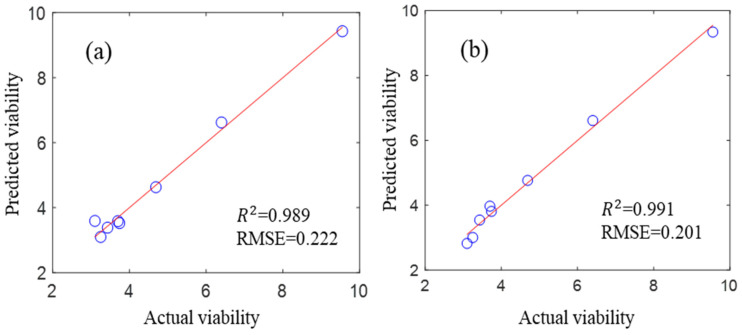
The performance of the trained class: (**a**) Vis/NIR-based and (**b**) NIR-based PLS models.

The performance of the built model for predicting the overall viability of mung bean seeds was also tested and evaluated. The accuracy difference in the models based on the two spectrometers was very small in the trained class (Figure 5). The *R*^2^ of the PLS models based on the Vis/NIR and NIR spectra were 0.989 and 0.991, respectively, and the RMSEs were 0.222 and 0.201, respectively.

The prediction deviation of seed viability (*PDSV*) was used as an index for evaluating the accuracy of the predicted class, and the results are shown in Table 4. The *PDSV* value of the PLS model based on the Vis/NIR spectrum was 20.71%, and the *PDSV* value of the PLS model based on the NIR spectrum was 21.65%, indicating that the Vis/NIR-based model was better than the NIR-based model. In addition, it was noted that the predicted value was lower than the real value when the seed viability was low, while the predicted value was higher than the real value when the seed viability was high. The results indicate that the developed model is practical and more likely to abandon batches of seeds with low viability, thus avoiding low germination rate and yield reduction caused by sowing low-viability seeds. Furthermore, it is easy to misjudge seeds with high viability as low-viability seeds and eliminate them, preventing the waste of seeds by culling them.

### 3.5. Design and Economic Assessment of the Automatic Mung Bean Seed System

**Figure 6:**
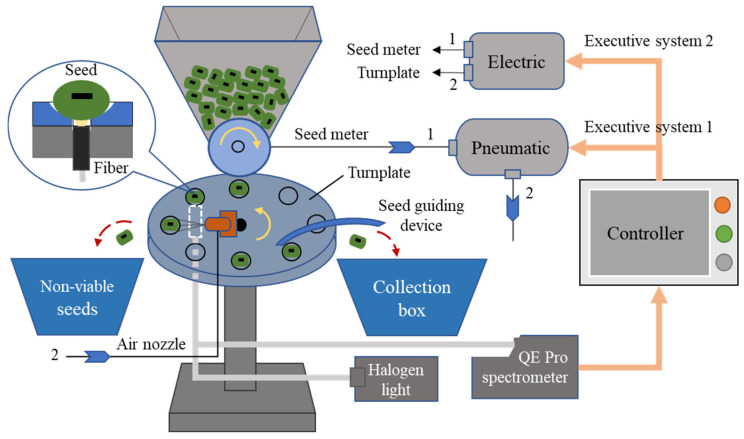
The automatic Vis/NIR-based system for the detection of seed viability and separation of non-viable seed of the mung bean.

An automatic Vis/NIR-based system for mung bean seeds was designed (Figure 6), and it was used for detecting seed viability and rejecting non-viable seeds in a large number of mung bean seeds. The system was composed of a spectral detection module (a QE Pro spectrometer, a halogen light source, an optical fiber, etc.), an electronic control module (motors, motor driver, speed reducer, etc.), a pneumatic control system (blower fan, air pump, valves, etc.), a set of mechanical mechanisms (seed meter, turnplate, rack, etc.), and a controller. The seed meter was used to obtain single seeds, which applied a pneumatic system to avoid seed damage in the process of testing. The sample holders were evenly arranged on the turnplate, and their rotational speed was adjusted according to the speed of the seed meter to ensure that all seeds were accurately put into the corresponding holders. The two-in-one spectral probe was fixed beneath the turnplate, and then the spectra of mung bean seeds were collected by the QE Pro spectrometer. For the function of seed viability prediction, the spectral data of 600 mung bean seeds (three groups with 200 seeds each) were continuously collected, and then the seed viability (average viability of the three groups) was predicted by the PLS model, which is an alternative to the traditional germination test with the advantages of speed and non-destructiveness. For the function of non-viable seed identification, the spectra of all individual seeds were collected, and then non-viable seeds were distinguished and separated by an air nozzle; the normal seeds were delivered into a collection box by a seed-guiding device, thus improving the emergence rate.

The lifespan of the Vis/NIR-based system was designed to be 10 years and could run for 29,200 h with 8 h a day. Its detection efficiency was 0.2 s/seed, and each test took 2 min for 600 seeds. Hence, the total power consumption for a test of seed viability is 0.05 kWh^−1^ (power 1.49 Kw, time 2 min). If the seed viability was measured by the germination test, the climate chamber worked 24 h a day, and 7 days were taken to complete the test; thus, a power of 50.4 kWh^−1^ was consumed (power 1.2 Kw, power ratio 0.25), which was much higher than the energy consumption of the spectral method at 0.05 kWh^−1^. Therefore, the developed Vis/NIR-based system is energy-efficient in seed viability detection.

The test costs of the two methods were compared, mainly including energy cost, equipment usage, labor cost, and seed consumption, which is shown in Table 5. The industrial charge in Beijing is $0.13 kWh^−1^. The energy cost of the spectral method is $0.01 per test, which is much lower than that of the germination test at $6.55. Although the equipment cost ($19,454.09) is higher than the cost of the germination test ($4750.6), the equipment usage cost of $0.02 is lower than that of the germination test at $27.33. This is because the running time of the spectral method is just 2 min while the time of the germination test lasts 7 days; thus, the equipment usage cost of the system is lower. The human labor costs are $6.59/h. In the 7-day period of the germination test, 1 h per day was needed to manage the seedlings, and the total labor cost was $46.1, which is higher than that of the spectral method ($0.22). The seeds used in the germination test cannot be recycled for sowing again, resulting in a seed consumption cost of $0.21 (the price of mung bean seeds for sowing is $6.32/kg, and their thousand kernel weight is 0.055 kg). The tested seeds by the spectral method can be reused for sowing due to their non-destructive nature. As a result, the total cost of the spectral method is $0.25, which is much lower than the cost of the germination test ($80.2). Therefore, the developed Vis/NIR-based system is economically sustainable in seed viability detection.

The Vis/NIR-based system can also identify and then separate non-viable seeds from mung bean seed batches. The cost for sorting non-viable seeds is shown in Table 6 (on the scale of one hectare of mung beans). The sowing amount of mung bean seeds is 18.75 kg per hectare, and 340,909 seeds need to be sown (thousand kernel weight of 55 g). The seed emergence rate and final crop yield can be increased by *R*_non_ when the *R*_non_ rate of non-viable seeds is eliminated, resulting in the revenue increase of $2223.8∙*R*_non_ (the hectare yield of mung bean is 1322.9 kg, and the price of harvested mung bean seeds is $1.68). The elimination of non-viable seeds increases the seed input by $118.56 *R*_non_/(1 − *R*_non_). In addition, the cost of energy consumption and equipment usage is $16.29/(1 − *R*_non_), and the labor cost is $6.59 for 1 h to operate the machine. Ultimately, the total revenue *R*_total_ is obtained, whose numerator is a quadratic equation of one variable with an opening facing down (Table 6). It shows that more than 1.1% of non-viable seeds being screened out will positively increase revenue, and the revenue of $81.22 per hectare can be increased at an *R*_non_ of 5%. When the seed germination rate is as low as 85% (the minimum germination rate for sowing, *R*_non_ = 15%), the profit can be increased to $286.9 per hectare due to the non-viable seeds being eliminated and the ultimate seed germination rate being improved.

## 4. Conclusions

This study discussed the potential of using QE Pro (185~1100 nm) and NIR Quest (900~2500 nm) spectrometers to predict seed viability and identify non-viable seeds of mung bean. The effects of different optimal spectral bands and modeling algorithms on the detection accuracy of seed viability were analyzed. Compared to the SVM and ELM algorithms, the PLS model coupled with the Vis/NIR spectrum had the highest accuracy, precision, and recall for the identification of non-viable seeds. Furthermore, the developed model could predict the viability of seed batches and identify individual non-viable seeds by non-destructive and environmentally friendly methods. According to the economic analysis, the automatic sorting system utilizing the technology of the Vis/NIR spectrum is economically viable with high efficiency, low energy consumption, and a large capacity to increase the germination rate of mung beans. There is no chemical solution added in the process, which promotes the cleaner production of mung beans.

Nevertheless, this work has several limitations: (1) The study used microwave-treated seeds to simulate viability loss; while validated with naturally aged seeds, broader field validation under diverse natural degradation conditions is needed. (2) The economic analysis assumes ideal operating conditions; actual costs may vary with regional electricity prices, labor costs, and equipment maintenance requirements. (3) The current system design is crop-specific for mung bean seeds; adaptation to other seed types requires mechanical modifications. To enhance model stability and generalizability, future research should: (1) expand the sample database to include seeds from multiple growing seasons, storage conditions, and natural aging processes; (2) integrate RGB imaging for simultaneous detection of multiple quality attributes (cracks, diseases, color defects) alongside viability, and investigate the effects of seed scratches, color variations, and shape abnormalities on detection accuracy; and (3) explore transfer learning approaches to adapt the model to other legume crops with minimal retraining.

## Figures and Tables

**Table 1:** The information on the collected mung bean seeds.

Number	Seed Variety	Producing Place	Average Size		Abbreviated Variety Name
			Length/mm	Width/mm	
1	JiLv2	Baoding City, Hebei Province	5.39 ± 0.34	4.11 ± 0.14	JL2
2	LiaoLv12	Shenyang City, Jilin Province	5.49 ± 0.29	3.91 ± 0.22	LL12
3	WanKeLv3	Hefei City, Anhui Province	5.00 ± 0.25	3.97 ± 0.12	WKL3
4	ZhongLv5	Hefei City, Anhui Province	5.54 ± 0.28	4.02 ± 0.20	ZL5
5	YingGe5	Zhangjiakou City, Hebei Province	4.82 ± 0.36	3.63 ± 0.30	YG5
6	YuLv1	Yulin, Shanxi Province	5.42 ± 0.26	4.17 ± 0.27	YL1
7	LvFeng2	Qiqihar City, Heilongjiang Province	4.21 ± 0.23	3.35 ± 0.23	LF2
8	BingLv20	Taiyuan City, Shanxi Province	5.63 ± 0.40	4.33 ± 0.18	BL20
9	JiLv16	Baoding City, Hebei Province	5.32 ± 0.24	4.17 ± 0.17	JL16
10	LiaoLv13	Shenyang City, Jilin Province	5.40 ± 0.36	3.94 ± 0.17	LL13
11	LvFeng5	Qiqihar City, Heilongjiang Province	5.30 ± 0.37	4.16 ± 0.17	LF5
12	BingLv21	Taiyuan City, Shanxi Province	6.03 ± 0.31	4.46 ± 0.18	BL21

**Table 2:** The results of the germination test of 12 mung bean varieties in normal groups.

Varieties	JL2	LL12	WKL3	ZL5	YG5	YL1	LF2	BL20	JL16	LL13	LF5	BL21
Sample number	200	200	200	200	200	200	200	200	200	200	200	200
Germination number	193	197	182	184	197	171	197	197	193	193	194	193
Germination rate (%)	96.5	98.5	91	92	98.5	85.5	98.5	98.5	96.5	96.5	97	96.5
Average length (cm)	3.88	3.15	3.77	5.1	6.495	3.8	9.695	3.76	3.025	3.075	4.865	3.17
Viability	3.74	3.1	3.43	4.69	6.4	3.25	9.55	3.7	2.92	2.97	4.72	3.06

**Table 3:** The accuracy of trained and predicted classes by the three algorithms (PLS, SVM, and ELM).

Algorithm	Trained Class			Predicted Class		
	Sample Number	Vis/NIR	NIR	Sample Number	Vis/NIR	NIR
		Accuracy/%	Accuracy/%		Accuracy/%	Accuracy/%
PLS	3200	98.9	98.5	1600	98.8	97.7
SVM	3200	99.9	100	1600	98.3	97.4
ELM	3200	98.7	95.1	1600	98.1	94.7

**Table 4:** The performance of the predicted class for seed viability.

Seed Varieties	Ture Viability	Vis/NIR			NIR		
		Predicted Value	*PDSV*/%	Total/%	Predicted Value	*PDSV*/%	Total/%
JL16	2.92	2.09	28.42	20.71	3.72	27.4	21.65
LL13	2.97	2.49	16.16		2.36	20.54	
LF5	4.72	5.4	14.41		4.94	4.66	
BL21	3.06	2.33	23.86		2.02	33.99	

**Table 5:** The cost for the viability determination of mung bean seed by spectroscopic method and germination test (sample size of 600 seeds).

Name	Spectral Method	Germination Test
Energy cost	$\begin{array}{ll} P_{1} & =P_{spectra}+P_{electric}+P_{Pneumatic}+P_{controller}\\ & =\left(0.01+0.26+1.2+0.02\right)\;kw=1.49\;kW \end{array}$ $t_{runing1}=600\;\mathrm{seed}\times 0.2\;s\cdot {seed}^{-1}=\frac{1}{30}h$ $C_{energy1}=1.49\;kW\times \frac{1}{30}h\times \$0.13{kWh}^{-1}=\$0.01$	$\begin{array}{ll} P_{2} & =P_{incubator\;}\times 0.25\\ & =1.2\;kW\times 0.25=0.3\;kW \end{array}$ $\begin{array}{ll} t_{runing2} & =7\times 24\;h=168\;h \end{array}$ $\begin{array}{ll} C_{energy2} & =0.3\times 168\times \$0.13\\ & =\$6.55 \end{array}$
Equipment usage cost	$\begin{array}{ll} E_{1} & =E_{spectra}+E_{electric}+E_{Pneumatic}+E_{controller}+E_{mechanical}\\ & =\$17955.7+\$218.12+\$410.95+\$316.12+\$553.20\\ & =\$19454.09 \end{array}$ $t_{life}=10\times 365\times 8h=29200h$ $\begin{array}{ll} C_{equip1} & =E_{1}\times t_{runing1}÷t_{life}\\ & =\$19454.09\times \frac{1}{30}÷29200=\$0.02 \end{array}$	$\begin{array}{ll} E_{2} & =E_{incubator}+E_{germ\;bed}\\ & =\$4742+\$8.6=\$4750.6 \end{array}$ $\begin{array}{ll} C_{equip1} & =E_{2}\times t_{runing2}÷t_{life}\\ & =\$4750.6\times 168÷29200\\ & =\$27.33 \end{array}$
Labor cost	$\begin{array}{ll} C_{labor1} & =C_{hour}\times t_{runing1}\\ & =\$6.59h^{-1}\times \frac{1}{30}h=\$0.22 \end{array}$	$\begin{array}{ll} C_{labor2} & =L_{hour}\times 7\left(day\right)\times 1h\\ & =\$6.59\times 7=\$46.1 \end{array}$
Seed consumption	$C_{seed1}=\$0\;$ The seeds can be used for sowing after testing	$\begin{array}{ll} C_{seed2} & =600\;seed\times W_{kg}\times S_{sow}\\ & =0.6\times 0.055\times \$6.32\\ & =\$0.21 \end{array}$
Total	$C_{total1}=\$0.25$	$C_{total2}=\$80.2$

**Table 6:** The detailed calculations about the revenue of identifying and eliminating non-viable seeds.

Name	Calculative Process
Benefit from the yield increase	$\begin{array}{ll} B_{yield} & =Y_{ha}\times S_{harvest}\times R_{non}\\ & =1322.9\;kg\times \$1.68\;{kg}^{-1}\times R_{non}=\$2223.8\cdot R_{non} \end{array}$
Cost for non-viable seed elimination	$\begin{array}{ll} C_{sow} & =\frac{Q_{ha}}{1000}\times \frac{R_{non}}{1-R_{non}}\times W_{kg}\times S_{sow}\\ & =\frac{340908}{1000}\times 0.055\;kg\times \$6.32\;{kg}^{-1}\times \frac{R_{non}}{1-R_{non}}\\ & =\$118.56\cdot \frac{R_{non}}{1-R_{non}} \end{array}$
Cost for machine usage	$\begin{array}{ll} t_{runing} & =340909\;\mathrm{seed}\times \frac{1}{1-R_{non}}\times 0.2\;s\cdot {\mathrm{seed}}^{-1}=\frac{18.94}{1-R_{non}}h\\ C_{machine} & =C_{energe}+C_{equip}+C_{labor}\\ & =1.49\;kw\times t_{runing}\times \$0.13\;{kWh}^{-1}+\$19454.09\times t_{runing}÷29200+\$6.586\;h^{-1}\times 1\;h\\ & =0.86t_{runing}+\$6.59=\frac{\$16.29}{1-R_{non}}+\$6.59 \end{array}$
Total revenue	$\begin{array}{ll} R_{total} & =B_{yield}-C_{sow}-C_{machine}\\ & =\frac{1}{1-R_{non}}(-\$2223.80{R_{non}}^{2}+\$2111.83R_{non}-\$22.87) \end{array}$

## Data Availability

The data used in this study were self-tested and self-collected. As the control method designed in this paper is still being further improved, data cannot be shared at present.

## References

[1] Zhou Q., Wang L., Zhang Y., Zhang C., Kong X., Hua Y., Chen Y. (2024). Characterization of mung bean endogenous proteases and globulins and their effects on the production of mung bean protein. Food Chem..

[2] Hou D., Yousaf L., Xue Y., Hu J., Wu J., Hu X., Feng N., Shen Q. (2019). Mung bean (*Vigna radiata* L.): Bioactive polyphenols, polysaccharides, peptides, and health benefits. Nutrients.

[3] Yi-Shen Z., Shuai S., Fitzgerald R. (2018). Mung bean proteins and peptides: Nutritional, functional and bioactive properties. Food Nutr. Res..

[4] Weishaar C.E., Phippen W.B. (2024). Indigo (*Indigo suffruticosa* Mill.) seed germination to improve commercial viability. Ind. Crops Prod..

[5] Norman P.E., Danquah A., Asfaw A., Tongoona P.B., Danquah E.Y., Asiedu R. (2020). Seed Viability, Seedling Growth and Yield in White Guinea Yam. Agronomy.

[6] Baek I., Kusumaningrum D., Kandpal L., Lohumi S., Mo C., Kim M., Cho B.-K. (2019). Rapid Measurement of Soybean Seed Viability Using Kernel-Based Multispectral Image Analysis. Sensors.

[7] He Y., Cheng J., He Y., Yang B., Cheng Y., Yang C., Zhang H., Wang Z. (2019). Influence of isopropylmalate synthase Os IPMS 1 on seed vigour associated with amino acid and energy metabolism in rice. Plant Biotechnol. J..

[8] Fu H., Cao D.-D., Hu W.-M., Guan Y.-J., Fu Y.-Y., Fang Y.-F., Hu J. (2017). Studies on optimum harvest time for hybrid rice seed. J. Sci. Food Agric..

[9] Wang X., Zheng H., Tang Q. (2018). Early harvesting improves seed vigour of hybrid rice seeds. Sci. Rep..

[10] Huang Y., Wu W., Zhao T., Lu M., Wu H., Cao D. (2021). Drying temperature regulates vigor of high moisture rice seeds via involvement in phytohormone, ROS, and relevant gene expression. J. Sci. Food Agric..

[11] Huang Y., Wu W., Zou W., Wu H., Cao D. (2020). Drying temperature affects rice seed vigor via gibberellin, abscisic acid, and antioxidant enzyme metabolism. J. Zhejiang Univ.-Sci. B.

[12] Wang P., Li D., Wang L., Adhikari B. (2017). Effect of High Temperature Intermittent Drying on Rice Seed Viability and Vigor. Int. J. Food Eng..

[13] De Vitis M., Hay F.R., Dickie J.B., Trivedi C., Choi J., Fiegener R. (2020). Seed storage: Maintaining seed viability and vigor for restoration use. Restor. Ecol..

[14] Lotito S., Quagliotti L. (1993). The influence of storage temperature and moisture content on seed viability in pepper (*Capsicum annuum* L). Agronomie.

[15] Thirusendura Selvi D., Saraswathy S. (2018). Seed viability, seed deterioration and seed quality improvements in stored onion seeds: A review. J. Hortic. Sci. Biotechnol..

[16] Branco M., Branco C., Merouani H., Almeida M.H. (2002). Germination success, survival and seedling vigour of Quercus suber acorns in relation to insect damage. For. Ecol. Manag..

[17] Turner R.E., Ebelhar M.W., Wilkerson T., Bellaloui N., Golden B.R., Irby J.T., Martin S. (2020). Effects of Purple Seed Stain on Seed Quality and Composition in Soybean. Plants.

[18] Alongi J., Costantini A., Ferruti P., Ranucci E. (2022). Evaluation of the eco-compatibility of polyamidoamines by means of seed germination test. Polym. Degrad. Stab..

[19] Muniyappan V.K., Tamilmani E., Desikan R., Ranganathan U. (2019). Influence of groundnut seed viability on biodiesel feedstock quality. Ind. Crops Prod..

[20] Lopez Del Egido L., Navarro-Miró D., Martinez-Heredia V., Toorop P.E., Iannetta P.P.M. (2017). A Spectrophotometric Assay for Robust Viability Testing of Seed Batches Using 2,3,5-Triphenyl Tetrazolium Chloride: Using *Hordeum vulgare* L. as a Model. Front. Plant Sci..

[21] Pereira D.F., Bugatti P.H., Lopes F.M., de Souza A.L.S.M., Saito P.T.M. (2021). Assessing Active Learning Strategies to Improve the Quality Control of the Soybean Seed Vigor. IEEE Trans. Ind. Electron..

[22] Zhang W., Li C., Zhou M. (2017). Methods for Crop Phenotypic Research.

[23] He X., Zhu J., Li P., Zhang D., Yang L., Cui T., Zhang K., Lin X. (2024). Research on a Multi-Lens Multispectral Camera for Identifying Haploid Maize Seeds. Agriculture.

[24] Yin H., Xie B., Chen B., Ma J., Chen J., Zhou Y., Han X., Xiong Z., Yu Z., Huang F. (2023). Detection of moisture content and size of pumpkin seeds based on hyperspectral reflection and transmission imaging techniques. J. Food Compos. Anal..

[25] He X., Liu L., Liu C., Li W., Sun J., Li H., He Y., Yang L., Zhang D., Cui T. (2022). Discriminant analysis of maize haploid seeds using near-infrared hyperspectral imaging integrated with multivariate methods. Biosyst. Eng..

[26] Liu S., Li L., Fan H., Guo X., Wang S., Lu J. (2020). Real-time and multi-stage recommendations for nitrogen fertilizer topdressing rates in winter oilseed rape based on canopy hyperspectral data. Ind. Crops Prod..

[27] Stenberg B., Viscarra Rossel R.A., Mouazen A.M., Wetterlind J. (2010). Visible and Near Infrared Spectroscopy in Soil Science. Advances in Agronomy.

[28] Wang Z., Fan S., An T., Zhang C., Chen L., Huang W. (2024). Detection of Insect-Damaged Maize Seed Using Hyperspectral Imaging and Hybrid 1D-CNN-BiLSTM Model. Infrared Phys. Technol..

[29] Feng L., Zhu S., Liu F., He Y., Bao Y., Zhang C. (2019). Hyperspectral imaging for seed quality and safety inspection: A review. Plant Methods.

[30] He Y., Liu F., Li X., Shao Y. (2015). Spectroscopy and Imaging Technology in Agriculture.

[31] Nogales-Bueno J., Rodríguez-Pulido F.J., Heredia F.J., Hernández-Hierro J.M., Baca-Bocanegra B. (2024). Use of hyperspectral imaging devices for the measurement of small granular samples: Evaluation of grape seed protein concentrates. LWT.

[32] Fassio A.S., Restaino E.A., Cozzolino D. (2015). Determination of oil content in whole corn (*Zea mays* L.) seeds by means of near infrared reflectance spectroscopy. Comput. Electron. Agric..

[33] Zhang L., Wang Y., Wei Y., An D. (2022). Near-infrared hyperspectral imaging technology combined with deep convolutional generative adversarial network to predict oil content of single maize kernel. Food Chem..

[34] Bilal M., Xiaobo Z., Arslan M., Tahir H.E., Azam M., Junjun Z., Basheer S., Abdullah (2020). Rapid determination of the chemical compositions of peanut seed (*Arachis hypogaea*) Using portable near-infrared spectroscopy. Vib. Spectrosc..

[35] Qiao M., Xu Y., Xia G., Su Y., Lu B., Gao X., Fan H. (2022). Determination of hardness for maize kernels based on hyperspectral imaging. Food Chem..

[36] Caporaso N., Whitworth M.B., Grebby S., Fisk I.D. (2018). Non-destructive analysis of sucrose, caffeine and trigonelline on single green coffee beans by hyperspectral imaging. Food Res. Int..

[37] Choi J.-Y., Heo S., Bae S., Kim J., Moon K.-D. (2020). Discriminating the origin of basil seeds (*Ocimum basilicum* L.) using hyperspectral imaging analysis. LWT.

[38] Huang M., Tang J., Yang B., Zhu Q. (2016). Classification of maize seeds of different years based on hyperspectral imaging and model updating. Comput. Electron. Agric..

[39] Shrestha S., Knapič M., Žibrat U., Deleuran L.C., Gislum R. (2016). Single seed near-infrared hyperspectral imaging in determining tomato (*Solanum lycopersicum* L.) seed quality in association with multivariate data analysis. Sens. Actuators B Chem..

[40] Ambrose A., Lohumi S., Lee W.H., Cho B.K. (2016). Comparative nondestructive measurement of corn seed viability using Fourier transform near-infrared (FT-NIR) and Raman spectroscopy. Sens. Actuators B Chem..

[41] Wakholi C., Kandpal L.M., Lee H., Bae H., Park E., Kim M.S., Mo C., Lee W.H., Cho B.K. (2018). Rapid assessment of corn seed viability using short wave infrared line-scan hyperspectral imaging and chemometrics. Sens. Actuators B Chem..

[42] Wang Y., Peng Y., Qiao X., Zhuang Q. (2021). Discriminant analysis and comparison of corn seed vigor based on multiband spectrum. Comput. Electron. Agric..

[43] Jin B., Qi H., Jia L., Tang Q., Gao L., Li Z., Zhao G. (2022). Determination of viability and vigor of naturally-aged rice seeds using hyperspectral imaging with machine learning. Infrared Phys. Technol..

[44] Wu N., Weng S., Chen J., Xiao Q., Zhang C., He Y. (2022). Deep convolution neural network with weighted loss to detect rice seeds vigor based on hyperspectral imaging under the sample-imbalanced condition. Comput. Electron. Agric..

[45] Fan Y., Ma S., Wu T. (2020). Individual wheat kernels vigor assessment based on NIR spectroscopy coupled with machine learning methodologies. Infrared Phys. Technol..

[46] Zhang T., Wei W., Zhao B., Wang R., Li M., Yang L., Wang J., Sun Q. (2018). A Reliable Methodology for Determining Seed Viability by Using Hyperspectral Data from Two Sides of Wheat Seeds. Sensors.

[47] Shrestha S., Deleuran L.C., Gislum R. (2017). Separation of viable and non-viable tomato (*Solanum lycopersicum* L.) seeds using single seed near-infrared spectroscopy. Comput. Electron. Agric..

[48] Yasmin J., Raju Ahmed M., Lohumi S., Wakholi C., Kim M., Cho B.-K. (2019). Classification Method for Viability Screening of Naturally Aged Watermelon Seeds Using FT-NIR Spectroscopy. Sensors.

[49] Kandpal L.M., Lohumi S., Kim M.S., Kang J.S., Cho B.K. (2016). Near-infrared hyperspectral imaging system coupled with multivariate methods to predict viability and vigor in muskmelon seeds. Sens. Actuators B Chem..

[50] Mukasa P., Wakholi C., Mohammad A.F., Park E., Lee J., Suh H.K., Mo C., Lee H., Baek I., Kim M.S. (2020). Determination of the viability of retinispora (*Hinoki cypress*) seeds using shortwave infrared hyperspectral imaging spectroscopy. J. Near Infrared Spectrosc..

[51] Hong S.J., Park S., Lee A., Kim S.Y., Kim E., Lee C.H., Kim G. (2023). Nondestructive prediction of pepper seed viability using single and fusion information of hyperspectral and X-ray images. Sens. Actuators A Phys..

[52] Liu J., Zhang D., Yang L., Ma Y., Cui T., He X., Du Z. (2022). Developing a generalized vis-NIR prediction model of soil moisture content using external parameter orthogonalization to reduce the effect of soil type. Geoderma.

[53] Liu S., Chen Z., Jiao F. (2023). Detection of maize seed germination rate based on improved locally linear embedding. Comput. Electron. Agric..

[54] Li J., Li C., Liao Q., Xu Z. (2019). Environmentally-friendly technology for rapid on-line recycling of acrylonitrile-butadiene-styrene, polystyrene and polypropylene using near-infrared spectroscopy. J. Clean. Prod..

[55] Noor P., Khanmohammadi M., Roozbehani B., Yaripour F., Bagheri Garmarudi A. (2018). Determination of reaction parameters in methanol to gasoline (MTG) process using infrared spectroscopy and chemometrics. J. Clean. Prod..

[56] Wu X., Li J., Yao L., Xu Z. (2020). Auto-sorting commonly recovered plastics from waste household appliances and electronics using near-infrared spectroscopy. J. Clean. Prod..

[57] Polder G., Blok P.M., de Villiers H.A.C., van der Wolf J.M., Kamp J. (2019). Potato Virus Y Detection in Seed Potatoes Using Deep Learning on Hyperspectral Images. Front. Plant Sci..

[58] Cui Y., Ge W., Li J., Zhang J., An D., Wei Y. (2019). Screening of maize haploid kernels based on near infrared spectroscopy quantitative analysis. Comput. Electron. Agric..

